# A molecular and preclinical comparison of the PD-1–targeted T-cell checkpoint inhibitors nivolumab and pembrolizumab

**DOI:** 10.1053/j.seminoncol.2017.06.002

**Published:** 2017-04

**Authors:** Petros Fessas, Hassal Lee, Shinji Ikemizu, Tobias Janowitz

**Affiliations:** a School of Clinical Medicine, University of Cambridge, Addenbrooke’s Hospital, Cambridge, United Kingdom; b MRC Laboratory of Molecular Biology, Cambridge, United Kingdom; c Division of Structural Biology, Graduate School of Pharmaceutical Sciences, Kumamoto University, Kumamoto, Japan; d Cancer Research UK Cambridge Institute, Li Ka Shing Centre, University of Cambridge, Cambridge, United Kingdom; e Department of Oncology, University of Cambridge, Cambridge Biomedical Research Centre and Addenbrooke’s Hospital, Cambridge, United Kingdom

**Keywords:** Nivolumab, Pembrolizumab, PD-1, T-cell checkpoint

## Abstract

T-cell checkpoint inhibition has a profound impact on cancer care and the programmed cell death protein 1 (PD-1)–targeted antibodies nivolumab and pembrolizumab have been two of the lead molecules of this therapeutic revolution. Their clinical comparability is a highly relevant topic of discussion, but to a significant degree is a consequence of their molecular properties. Here we provide a molecular, preclinical, and early clinical comparison of the two antibodies, based on the available data and recent literature. We acknowledge the limitations of such comparisons, but suggest that based on the available data, differences in clinical trial outcomes between nivolumab and pembrolizumab are more likely drug-independent than drug-dependent.

## 1 Introduction

Every decision on drug therapy by oncologists is influenced by a set of parameters, such as the efficacy of the drug in the respective patient population, the side effects profile, and the pharmacology of the drug. In this issue of *Seminars in Oncology*, Vinay Prasad and Victoria Kaestner discuss the specific example of clinical evidence and decision-making for the administration of nivolumab and pembrolizumab, two recently developed anti-checkpoint monoclonal antibodies (mAbs) that target programmed cell death protein 1 (PD-1). Based on clinical data they argue that both drugs should be considered interchangeable. Such clinical data are ultimately the consequence of the interactions of a drug’s molecular behaviour with the host’s pathophysiology. In this review, we thus provide a molecular comparison of nivolumab and pembrolizumab to assess whether there are any drug-specific arguments against clinical interchangeability.

PD-1 is an inhibitory T-cell surface receptor that promotes self-tolerance by suppressing T-cell activation. On ligand binding by PD-L1 or PD-L2, the PD-1 receptor blocks signaling in T cells by recruiting a phosphatase, SHP-2, which dephosphorylates the antigen receptor expressed by these cells [1]. Both PD-L1 and PD-L2, but PD-L1 especially, are often overexpressed in tumor cells[2], while PD-1 is highly expressed on T cells in patient tumors [3]. In addition, tumor expression of PD-L1 and T-cell expression of PD-1 correlates with tumor aggressiveness and poor clinical outcome [4], [5], [6]. The high frequency of PD-1/PD-L1 axis overactivation in tumors and its correlation with poor patient prognosis identify this axis as a candidate target for mAb therapy.

Nivolumab and pembrolizumab are the first two anti–PD-1 mAbs that have received US Food and Drug Administration (FDA) approval. Each has eight total approved indications, four of which overlap and four of which are discordant. Nivolumab is uniquely approved for initial therapy with ipilimumab for melanoma [7]; although not yet published, comparable response rates have been shown with pembrolizumab [8]. Similarly, the approved response rate in urothelial cancer for nivolumab [9] is analogous to that of pembrolizumab, for which approval is pending [10]. Nivolumab is approved as second-line therapy for renal cell carcinoma [11], but there are no comparable data for pembrolizumab as of yet. The remaining discordant indication is that for metastatic non-small cell lung carcinoma, for which pembrolizumab has been approved but nivolumab was shown to be non-superior to chemotherapy [12], [13]. Such discrepancies may be due either to drug-dependent or -independent reasons. By conducting a comparison of the two antibodies at a molecular level, we address whether different trial outcomes were due to an inherent difference in their mechanisms of action or pharmacokinetic properties or if they are more likely due to the discrepancies in clinical trial design.

Given the profound impact that cancer immunotherapy is beginning to deliver and the rapid increase in the numbers of mAb checkpoint inhibitors being investigated and licensed in cancer therapy [14], [15], it is of increasing importance that we understand how interchangeable mAb inhibitors are likely to be when they share a common therapeutic target.

Another key consideration with regards to checkpoint inhibition and targeted mAb cancer therapy is the increasing need for a unified approach to identifying which patients have the correct target and therefore are most likely to respond to target inhibition. In the case of PD-1/PD-L1, most clinical studies look only at patient populations of a certain cancer type, leading to split labels with different anti–PD-1 antibodies for different cancer types. However, given the molecular properties of these drugs, basket trials that investigate efficacy of different PD-1 inhibitors across cancer patients independent of tumor site but dependent on their immunological status [16] or PD-1/PD-L1 expression levels may give us a more inclusive answer with regard to patient selection. At the same time, these trials would offer a better understanding of the interchangeability of the medications.

## 2 Proposed mechanism of action of anti–PD-1 antibodies

Therapeutic antibodies are excellent examples of the profound link between protein structure and function and it is our understanding of this relationship that has allowed the engineering of structural modifications that enhance antibody therapies. Antibodies can be functionally divided into a variable and constant region. The variable region of the antibody binds the target (in this case, the immune checkpoint molecule, PD-1). The high binding affinity and specificity of this interaction is responsible in large part for the clinical effectiveness of antibodies as therapeutic molecules. The binding of antibodies to surface receptors as targets can have numerous effects. It can directly neutralize receptor/ligand binding, but it can also induce internalization of the receptor from the cell surface [17]. Most therapeutic antibody classes contain two or more epitope binding sites, so they can mimic the dimerization event of cell surface receptors and exhibit agonist activity [18]. The constant region of the antibody can add further layers of drug action. Depending on the antibody class, the constant region can interact with various receptors of the immune system to recruit antibody-mediated immune effector functions such as target cell lysis or phagocytosis [19]. In addition, engineered drug adducts can be added to the antibody to enable targeted delivery of toxic molecules to tumor cells [20].

**Fig. 1:**
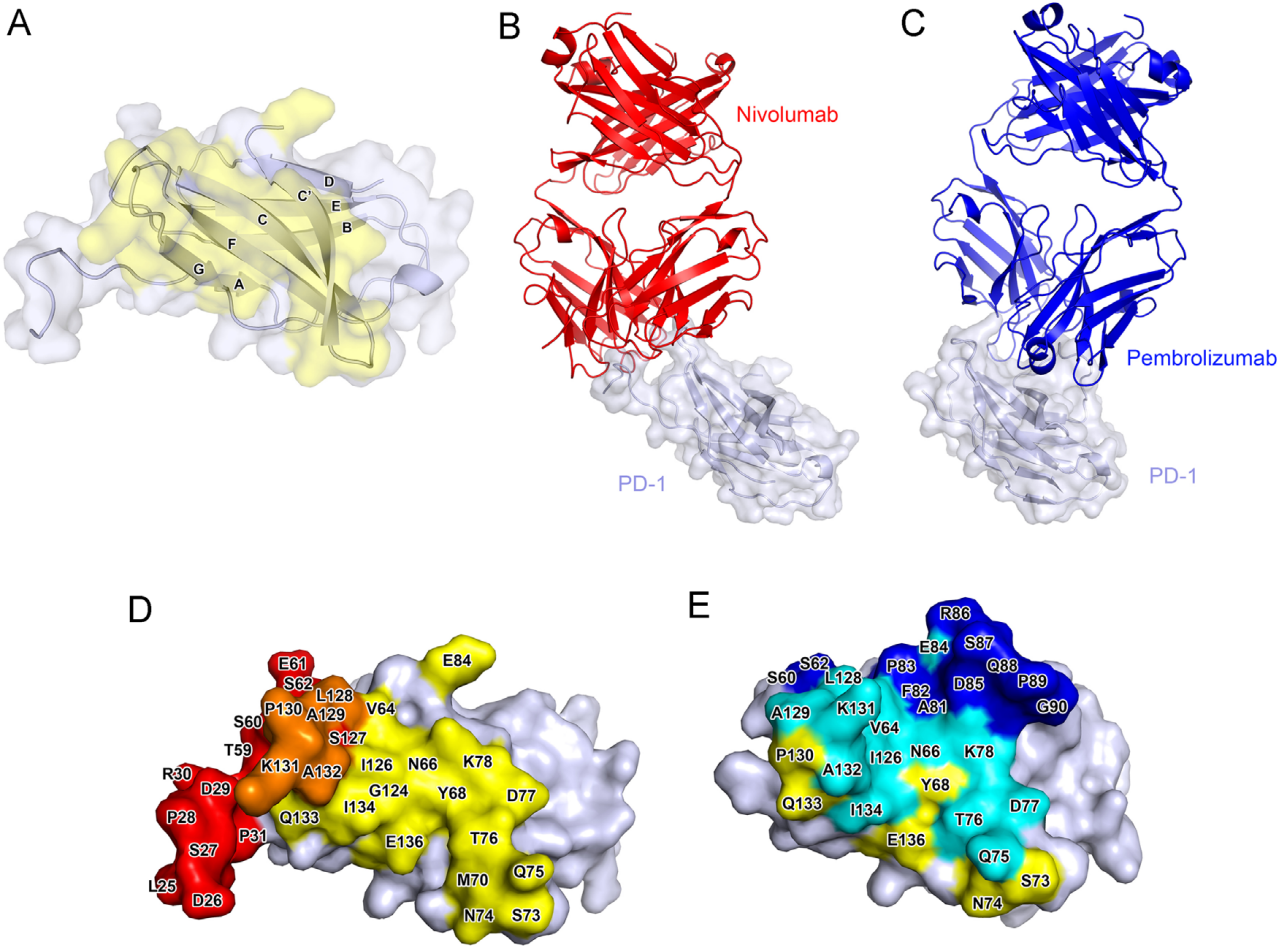
Structural comparison of nivolumab and pembrolizumab in complex with the extracellular domain of PD-1. (A) Ribbon diagram of the extracellular domain of PD-1. The molecular surface of PD-1 is represented in faint transparent blue, and the PD-L1 binding area in yellow. The (B) PD-1-nivolumab complex (PDB ID: 5WT9) and (C) PD-1-pembrolizumab complex (PDB ID: 5GGS) are shown as ribbon diagrams. Nivolumab and pembrolizumab are shown in red and blue, respectively. PD-1 in both complexes is drawn in light blue with transparent surfaces. (D) Surface representation of PD-1 from the PD-1-nivolumab complex. The PD-L1 and nivolumab binding areas on PD-1 are shown in yellow and red, respectively. The overlapping residues for binding both with PD-L1 and nivolumab are shown in orange. (E) Surface representation of PD-1 from the PD-1-pembrolizumab complex. The PD-L1 and pembrolizumab binding areas on PD-1 are shown in yellow and blue, respectively. The overlapping residues for binding both with PD-L1 and pembrolizumab are shown in cyan. (A), (D), and (E) are drawn with the same orientations. Amino acid single letter codes and primary sequence numbers are provided in (D) and (E). The differences in appearance in (A), (D), and (E) are a consequence of the fact that both antibodies stabilize different parts of the flexible PD-1 loop structures. Lack of stabilization of a flexible loop would impair the structural resolution by x-ray crystallography.

In the case of PD-1 inhibitors, the proposed mechanism of action is singular: the blockade of the PD-1-PD-L1/2 interaction. Both nivolumab and pembrolizumab target epitopes on the PD-1 molecule with high affinity and specificity [21], [22] (Fig. 1). They are both of the IgG4 subclass, which is by and large incapable of activation of host effector functions, as it only very weakly induces complement and cell activation due to low affinity for C1q and Fc receptors [23]. Given the same IgG4 subclass, an amino acid sequence comparison of the two antibodies shows that nivolumab and pembrolizumab are essentially identical apart from the variable regions that give rise to the paratopes—the variable regions that bind the epitope of the antigen (Table 1 and Fig. 1). It is therefore reasonable to expect any drug-dependent reasons for differences in their clinical efficacy to be ultimately secondary to the differences in the epitope–paratope binding. It is now possible to examine in detail the structural basis of epitope binding to elucidate the molecular mechanisms underlying the checkpoint blockade by these two antibodies.

**Table 1:** Comparison of the structural properties of nivolumab and pembrolizumab.

Antibody property	Nivolumab	Pembrolizumab	Expected effect on clinical outcome
Epitope	Binding is dominated by interactions with the PD-1 N-loop.	Binding dominated by interactions with PD-1 CD loop.	The epitope determines the drug’s molecular target and therefore its mode of action. Good choice of target is crucial for clinical efficacy and reducing on-target side effects.
	Total buried surface 1487-1932.5 Å^2^ (25,26)	Total buried surface 1774-2126 Å^2^ (26,27)	
Affinity	Affinity for recombinant human PD-1 protein (surface plasmon resonance): K_d_=3.06 pM(21)	Affinity for recombinant human PD-1 protein (ELISA): K_d_=29 pmol/L [22]	The strength with which antibody binds target molecule alters drug potency, dosing regimen and degree of on-target side effects.
Specificity	No binding to other members of superfamily: CD28, ICOS, CTLA-4, and BTLA (ELISA) [21].	No data found.	As antibody specificity decreases, off-target side effects become more likely.
Degree of humanization	Antibody generated in humanized mice containing human immunoglobulin minilocus for both the heavy chain and light chain kappa locus.	Antibody generated in humanized mice containing human immunoglobulin minilocus for both the heavy chain and light chain locus.	As the proportion of human sequences increase, immunogenicity of the drug decreases thus increasing drug stability and potency.
Antibody class	IgG4 subclass	IgG4 subclass	Affects avidity, mechanism of drug action (certain isotypes competent for antibody dependent cell cytotoxicity) and molecular size. Thus affects clinical efficacy, potency, and tissue penetration.
Glucose modifications	CHO production—no additional sugar modifications	CHO production—no additional sugar modifications	Fc region binding to immune receptors can be modulated by targeted sugar modifications. This can enhance mechanisms of action and affect drug stability.

Note.Comparison of the epitope, affinity, specificity, degree of humanization, antibody class and glucose modifications of nivolumab and pembrolizumab, alongside a discussion of the expected effect of these properties on clinical outcome.

PD-1 = programmed cell death protein 1; ELISA = enzyme-linked immunosorbent assay; CD28 = cluster of differentiation 28 protein; ICOS = inducible T-cell costimulator protein; CTLA-4 = cytotoxic T-lymphocyte–associated protein 4; BTLA = B- and T-lymphocyte attenuator protein; CHO = Chinese hamster ovary cell.

## 3 Comparison of the structural basis of PD-1 blockade by pembrolizumab and nivolumab

PD-1 is a type I transmembrane protein that spans the cell lipid bilayer once. Its ectodomain consists of the signal peptide, an N-terminal extension, a V-set immunoglobulin domain, and a stalk region (Fig. 1A). Solved crystal structures of the PD-1 receptor in complex with hPD-L1 enable us to visualize the native interaction between PD-1/PD-L1 which is mediated mostly by the residues of the C′CFG strands of both PD-1 and PD-L1. This interaction covers a buried surface area of 1,970 Å^2^ and induces the PD-1 CC′ loop to change in conformation slightly, enabling it to close around the PD-L1 molecule on binding [24].

Crystal structures of the PD-1 ectodomain in complex with the Fab fragments of nivolumab (Fig. 1B) and pembrolizumab (Fig. 1C) have shown that there is a significant overlap between the epitopes of both of these mAbs with the PD-L1 binding site. Nivolumab binds PD-1 by using the N-terminal extension, FG and BC loops as a platform for binding (Fig. 1B and 1D). The binding affinity is heavily dependent on the N terminal extension, which is not involved in PD-L1 recognition, while the overlapping binding surface shared by the V_L_ region of the antibody and PD-L1 resides mostly on the FG loop (Fig. 1D) [25]. On the other hand, the interaction of pembrolizumab with PD-1 is heavily dependent on the flexible C′D loop of PD-1, which is not involved in the interaction with PD-L1 (Fig. 1C and 1E) [26], [27]. However, its interaction also with the C and C′ strands of PD-1 ensure that it competes with the binding of PD-L1 (Fig. 1E). In addition, binding of either of these antibodies induces small conformational changes in the flexible BC and FG loops of PD-1, which are incompatible with PD-L1 binding (Fig. 1D and 1E) [25], [26]. These structural features suggest that both these antibodies have a similar mechanism of action whereby they competitively inhibit PD-L1 binding by direct occupancy and steric blockade of the PD-L1 binding site.

There are thus notable structural differences between the two antibodies in terms of how they bind to PD-1. The pembrolizumab epitope region shows a much greater overlap with the PD-L1 binding site than the epitope region of nivolumab. Strikingly, there is almost no overlap between the binding sites of pembrolizumab and nivolumab on the PD-1 molecule. In keeping with these findings, competitive binding analysis suggests that there is partial complementary binding between the two antibodies [25]. However, whether simultaneous administration of the two drugs would offer an improved therapeutic strategy is questionable given how well the antibodies block PD-1/PD-L1 binding. As single agents, the binding affinities of both antibodies to the recombinant human PD-1 measured by enzyme-linked immunosorbent assay (ELISA) or surface plasmon resonance are in a similar low picomolar range, as summarized in Table 1. A caveat to this comparison, however, is that the binding studies are likely to have been performed with bivalent antibodies in solution, rather than Fab fragments, which permits the measurement of affinity to be influenced by steric factors.

## 4 The preclinical and early clinical pharmacology of nivolumab and pembrolizumab are similar

With regard to biological assessment, both antibodies were initially tested in mixed lymphocyte reactions, where they were added to co-cultures of T cells, which express PD-1 when activated, together with allogeneic monocytes, which present PD-L1 on their surfaces [3]. While CTLA-4 and PD-1 were thought to be different in CTLA-4 effecting the T-cell priming phase and PD-1 inhibiting the effector function of activated T cells [3], recent data suggest that they both effect the CD28 pathway [28], [29]. In any case, cytokine production has been used as a key metric of response to anti-PD-1 antibodies. In mixed lymphocyte reactions, the cytokine monitored to demonstrate T-cell response in culture was different for the two antibodies, and therefore not directly comparable: interferon gamma (IFNγ) production from CD4^+^ T cells was used for nivolumab, while interleukin (IL)-2 production from Jurkat cells was used for pembrolizumab [21], [22]. Specifically, at concentration ranges of 0.05–50 μg/mL, nivolumab led to increases in IFNγ concentration of 1,000–4,000 pg/mL, while pembrolizumab at concentration ranges of 0.01–100 nmol/L (0.0149–149 μg/mL) led to IL-2 increases of 1,500–2,500 pg/mL. IFNγ and IL-2 are both fair choices, as PD-L1 engagement inhibits PI3K and its downstream target Akt kinase [30], which in turn upregulates both IL-2 and IFNγ [31]. In both cases, the responses were judged sufficient to move on to monitoring bona fide antigen-specific response.

Both antibodies were able to stimulate antigen-specific responses. When memory T cells were restimulated with cognate antigens, the presence of both antibodies enhanced IFNγ secretion [21], [22]. Specifically, when herpes virus C (HCV)-seropositive donor mononuclear cells were cultured with an HCV-specific antigen for 6 days and then restimulated, the presence of nivolumab was found to increase IFNγ production 2.3-fold by flow cytometry. In the biologically most closely resembling study performed with pembrolizumab, staphylococcus enterotoxin B was used to restimulate healthy human or cancer patient donor T cells. The IFNγ response was of a similar magnitude to nivolumab, and was similar for healthy (2.0-fold) and cancer patients (1.5-fold).

The assessment of both antibodies crucially also involved murine tumor models. Subcutaneous tumors derived from the MC38 colon adenocarcinoma line were assessed for both antibodies. As with in vitro studies, direct comparison is difficult due to diverging study designs. In the most analogous set of experiments, the antibodies were similarly effective. Doses of 10 mg/kg intraperitoneally given on days 7, 10, and 13 post-implantation for nivolumab led to growth inhibition of 76% at day 20, while the same dose for pembrolizumab given on days 6, 10, 13, 16, and 20 led to tumor growth inhibition of 92.5% at day 20 [21], [22]. Since neither nivolumab nor pembrolizumab recognize murine PD-1, surrogate anti-mouse PD-1 antibodies were used; the efficacy of surrogate antibodies suggests in itself that blockage by any antibody is likely to lead to response.

Following unremarkable preclinical toxicology studies, both antibodies were moved to early clinical studies to determine their human pharmacology. Receptor occupancy (RO) for nivolumab was studied in 15 patients using flow cytometric methods [32]. Mean peak occupancy was 85% at 4–24 hours, while mean plateau occupancy was 72% after 57 days, values consistent with the high in vitro affinity of nivolumab. Remarkably, RO was found to be dose-independent for the range tested (0.3–10 mg/kg), again reflecting the very high affinity of the antibody. The RO of pembrolizumab was indirectly calculated using ex vivo IL-2 stimulation; therefore, no comparable values are given. However, RO saturation was also reached after 1 mg/kg, which is very similar to nivolumab [33].

There was no significant change on the IFNγ Enzyme-Linked ImmunoSpot (ELISPOT) assay on blood samples from patients receiving either antibody, which indicates no compromise of the overall T-cell–mediated immune response [32], [33]. Finally, the clinical pharmacokinetics of the two antibodies are very similar, as summarized in Table 2.

**Table 2:** Pharmacokinetic properties of nivolumab and pembrolizumab [21], [22].

	Nivolumab	Pembrolizumab
Clearance	0.2 L/d via nonspecific catabolism	0.2 L/d via nonspecific catabolism
Terminal half-life	26.7 days	26 days
Steady-state concentrations	Reached by 12 weeks when administered at 3 mg/kg every 2 weeks (6 doses)	Reached by 18 weeks when administered at 2 mg/kg every 3 weeks (6 doses)
Recommended dose	3 mg/kg IV over 60 min every 2 weeks	2 mg/kg IV over 30 min every 3 weeks

Note. Comparison of the clearance, terminal half-life, recommended dose and steady-state concentrations of nivolumab and pembrolizumab.

IV = intravenous.

## 5 Conclusion

In this article, we compare the molecular, preclinical and early clinical characteristics of the PD-1–targeted T-cell checkpoint inhibitory antibodies nivolumab and pembrolizumab. The significant molecular similarities between these drugs suggest that differences observed in clinical data are unlikely to be drug-dependent, and are likely to be due to drug-independent differences. These may, for example, be due to differences between the patient populations in the clinical trials designed to test the drugs.

Both antibodies are of the IgG4 subclass; their binding relies heavily on interactions with the flexible loops of PD-1 and their epitopes include residues of the PD-L1 binding site. Although these overlaps are of different extent and in different spatial locations, both antibodies block the interaction of PD-1 with PD-L1 effectively.

For both antibodies, T-cell activation was shown. While the preclinical assessment protocols for the two antibodies are not directly comparable, inferences can be made due to similarities in the readouts. There are no indications of any significant differences from in vitro or animal experiments that would support a drug-dependent explanation for different indications. The early clinical pharmacokinetic data are also very similar.

Although such comparisons have limitations, given these molecular and preclinical assessments of nivolumab and pembrolizumab, it appears reasonable to postulate drug-dependent interchangeability. However, our current knowledge of drug design and cancer immunology holds a cautionary tale against such predictions based purely on preclinical data and on potentially incomplete mechanistic understanding. For example, different CD28 agonist antibodies with similar preclinical data showed extreme differences in clinical profiles [34]. Nevertheless, the pharmacological mechanism of antibody-mediated receptor agonism is arguably more complex than the blockade mechanism through which antibody-based receptor antagonism is thought to work. We acknowledge as well that preclinical comparisons of medications, including therapeutic antibodies, will not provide substitute for clinical assessment of toxicities, which are extremely important in guiding decision-making for patient management. However, the toxicity due to checkpoint blockade is mostly attributable to auto-immunity [35]. Since these complications are detected in studies on PD-1-knockout mice, they are likely to be attributable to on-target effects of the antibodies [36]. A further limitation of our manuscript is the focus on the PD-1/PD-L1 interaction. Other known or unknown ligands of PD-1 may be differently affected by the reported drugs. Lastly, we recognize that comparing two medications will not provide a general answer for a class of drugs and cannot substitute for in-depth analysis of every new agent. In view of these considerations, we also explicitly do not comment on the interchangeability of anti-PD1 and anti–PD-L1 therapy.

Our attempt to compare the preclinical evidence supporting the clinical use of nivolumab and pembrolizumab highlights a degree of nonconcordance of the reported data and thereby the data available to scientists and licensing agencies. Although the key aspects of pharmacodynamics and pharmacokinetics are addressed, we wonder whether the field should strive towards greater concordance when reporting the assessment of drugs from similar classes to facilitate academic dialogue. In other areas of medicine, such as cardiovascular practice, interchangeability of drugs within the same class has been reflected in clinical guidelines. This question should remain a focus as we develop immunotherapy in cancer.

In summary, the available molecular, preclinical, and early clinical data on nivolumab and pembrolizumab support the conclusions that both drugs may well be interchangeable. This in turn means that disparity of trial results is most likely due to drug-independent reasons, possibly related to trial design and patient selection [37], which should be carefully examined and are likely to be highly informative.

## Conflicts of interest

The authors report no conflict of interest. The drug names are in alphabetical order.
